# Use of Novel Antibiograms to Determine the Need for Earlier Susceptibility Testing and Administration for New β-Lactam/β-Lactamase Inhibitors in the United States

**DOI:** 10.3390/antibiotics11050660

**Published:** 2022-05-14

**Authors:** Kenneth P. Klinker, Levita K. Hidayat, Eric Wenzler, Joan-Miquel Balada-Llasat, Mary Motyl, C. Andrew DeRyke, Karri A. Bauer

**Affiliations:** 1 Merck & Co., Inc., 2000 Galloping Hill Rd., Kenilworth, NJ 07033, USA; kenneth.klinker@merck.com (K.P.K.); levita.hidayat@merck.com (L.K.H.); mary_motyl@merck.com (M.M.); andrew.deryke@merck.com (C.A.D.); 2 Department of Pharmacy Practice, College of Pharmacy, University of Illinois Chicago, 833 South Wood St., Room 164, Chicago, IL 60612, USA; wenzler@uic.edu; 3 Department of Clinical Microbiology and Clinical Pathology, The Ohio State University Wexner Medical Center, 181 Taylor Ave., Columbus, OH 43203, USA; joan.balada@osumc.edu

**Keywords:** syndromic antibiogram, susceptibility testing, Enterobacterales, *Pseudomonas aeruginosa*, extended-spectrum β-lactamase, cefepime, piperacillin/tazobactam, meropenem, ceftolozane/tazobactam, imipenem/relebactam

## Abstract

Antimicrobial resistance is a global public health threat, and gram-negative bacteria, such as Enterobacterales and *Pseudomonas aeruginosa,* are particularly problematic with difficult-to-treat resistance phenotypes. To reduce morbidity and mortality, a reduction in the time to effective antimicrobial therapy (TTET) is needed, especially among critically ill patients. The antibiogram is an effective clinical tool that can provide accurate antimicrobial susceptibility information and facilitate early antimicrobial optimization, decrease TTET, and improve outcomes such as mortality, hospital length of stay, and costs. Guidance is lacking on how to validate the susceptibility to new antibacterial agents. Commonly used traditional and combination antibiograms may not adequately assist clinicians in making treatment decisions. Challenges with the current susceptibility testing of new β-lactam/β-lactamase inhibitor combinations persist, impacting the appropriate antibacterial choice and patient outcomes. Novel antibiograms such as syndromic antibiograms that incorporate resistant gram-negative phenotypes and/or minimum inhibitory concentration distributions may assist in determining the need for earlier susceptibility testing or help define an earlier optimal use of the new β-lactam/β-lactamase inhibitors. The purpose of this review is to emphasize novel antibiogram approaches that are capable of improving the time to susceptibility testing and administration for new β-lactam/β-lactamase inhibitors so that they are earlier in a patient’s treatment course.

## 1. Introduction

Antimicrobial resistance is one of the greatest global public health challenges of our time, with deaths attributed to resistant infections projected to exceed 10 million per year by 2050 [1]. Gram-negative pathogens with difficult-to-treat resistance (DTR) phenotypes, including Enterobacterales, *Pseudomonas aeruginosa*, and *Acinetobacter baumannii*, are particularly problematic, as they are associated with increased mortality and the use of second-line agents with a high toxicity and low efficacy [1,2]. Ensuring optimal outcomes for patients infected with these DTR pathogens requires a multifaceted approach, including appropriate risk stratification, knowledge of local antimicrobial flora and resistance, and the integration of diagnostic tools and antimicrobial stewardship interventions [3].

The single most important modifiable risk factor for mortality in patients with resistant gram-negative infections is the time to effective antimicrobial therapy (TTET) [4,5,6]. Commonly used empiric antibacterial agents such as cefepime (FEP) and piperacillin/tazobactam (TZP) may not adequately provide coverage for DTR gram-negative pathogens, such as those producing extended-spectrum β-lactamase (ESBL) and/or carbapenemase enzymes [7]. Further, conventional microbiologic methods often require ≥72 h for pathogen identification and susceptibility reporting [8]. During this period, empiric antibacterial adjustments are often made in response to a patient’s clinical deterioration before or in the absence of documented susceptibility, especially when employing broad-spectrum β-lactams and β-lactam/β-lactamase inhibitors [9]. These therapeutic decisions are typically further impaired by an inadequate understanding of resistance patterns, including cross-resistance, site and/or infection-specific resistance rates, and clinical factors capable of predicting resistance.

Fortunately, there are a variety of tools available to clinicians in assisting with early antimicrobial optimization, decreasing TTET, and improving outcomes such as mortality, length of stay, and costs. One such tool is the antibiogram, which has evolved in its complexity and utility from its traditional version into more modern forms capable of providing more accurate and usable antimicrobial susceptibility information [10,11]. Data from these antibiograms may assist clinicians in making more informed and timely antibacterial susceptibility testing requests for new antibacterial agents for patients. The purpose of this review is to outline the challenges associated with the current susceptibility testing paradigm for new antibacterial agents and provide novel antibiogram approaches capable of improving the time to susceptibility testing and administration of the new β-lactam/β-lactamase inhibitors.

## 2. Current Challenges with Susceptibility Testing of New Antibacterial Agents

Patients infected with resistant gram-negative pathogens have limited treatment options; however, in recent years, several new β-lactam/β-lactamase inhibitors have been approved by the United States Food and Drug Administration (FDA) with activity against these bacteria [12]. While these antibacterial agents are needed additions to the antimicrobial armamentarium, clinicians must balance the appropriate versus unnecessary administration of novel agents to decrease the development of resistance. Additional important considerations on the use of new antibacterial agents are the availability and implementation logistics associated with susceptibility testing. Importantly, susceptibility testing for new agents may not be routinely available in institutions. A recent electronic survey of the American College of Clinical Pharmacy Infectious Diseases Practice and Research Network determined that 37/50 (74%) respondents in 28 states had an in-house microbiology laboratory; however, 26% had to send samples to a health system core laboratory or use a third-party reference laboratory [13]. Overall, only 30% of respondents reported that their respective microbiology laboratories did complete susceptibility testing for any new antibacterial agent, and most respondents reported a >96-h turnaround time before the receipt of results.

Clinical microbiology laboratories provide antimicrobial susceptibility testing (AST) using different methodologies that range from manual disc or gradient diffusion to broth microdilution and commercial automated antimicrobial susceptibility testing (cAST) [14]. Agencies such as the College of American Pathologists require laboratories to perform verification studies for testing any new antibacterial agent in the laboratory in accordance with Clinical Laboratory Improvement Amendments [15]. Verifying the susceptibility for a new antibacterial agent can be challenging, time-consuming, and costly. Typically, the performance data on new antibacterial agents for the cAST system is not available at the time when the antibacterial agent is approved; therefore, laboratories need to rely on verifying manual testing or sending the isolate to a reference laboratory.

Verifying AST is complicated, as pathogens with a known susceptibility are needed and as ideally some of these should harbor resistance mechanisms targeted by a new antibacterial agent and should test near the clinical minimum inhibitory concentration (MIC) breakpoint [16]. Importantly, when a new antibacterial agent receives FDA approval, manual susceptibility testing modalities may not be readily available, and automated susceptibility devices are typically not available for several years after approval. Guidance on how to validate the susceptibility of a new antibacterial agent is lacking, and laboratories need to rely on the FDA validation of AST procedures. The results may not be reproducible because of differences in the quality of the disk or gradient strip, lot-to-lot variability, or differences in the commercial media used to set up AST [14]. The process is further complicated if the reagents for testing are not commercially available or only available through manufacturers with “research use only” [15]. Additionally, an ongoing quality assurance program needs to be implemented alongside AST to ensure the routine investigation of unexpected resistance or susceptibility once new antibacterial AST is verified. AST should be confirmed by a comparator laboratory, and if resistance is observed, the isolates should be profiled; however, these may not be available options [15]. These challenges and the potential unavailability of susceptibility testing for new antibacterial agents may significantly impact an appropriate antibacterial drug selection and patient outcomes.

## 3. Antibiograms

One of the most important tools available in assisting with risk stratification and the optimization of empiric antibacterial therapy are antibiograms. Antibiograms provide region- or country-level guidance for appropriate empiric therapy based on resistance patterns within the patient population of an institution, region, or country, but data may not be generalizable to specific patient populations or locations [10,11]. The Clinical and Laboratory Standards Institute M39 provides guidance to microbiology laboratories for the development of an antibiogram to ensure accuracy, reliability, and statistical validity [17]. The guidance details components of an antibiogram such as the included pathogens, number of isolates, antibacterial agents evaluated, and percentage of each pathogen/antibacterial agent combination that is interpreted as susceptible based on MIC criteria. For patients in whom the risk of mortality or significant morbidity is high, including those with sepsis and patients in the intensive care unit (ICU), an antibacterial agent with a percent susceptibility of ≥90% or 95% should be selected [17,18].

There are a variety of antibiograms that can be used by clinicians to select an appropriate empiric antibacterial therapy. Commonly, institutions use traditional antibiograms because they are readily available, easily understood by clinicians, and can be quickly incorporated into disease-state treatment guidelines [11]. An example of the traditional antibiogram reporting is the percent of *P. aeruginosa* that is susceptible to single drugs listed, such as FEP, TZP, or meropenem (MEM). There are several limitations of traditional antibiograms, including the lack of inclusion of the infection source and/or hospital location, lack of incorporation of patient variables, and limited correlation with clinical and microbiologic outcomes [11]. Because of the associated limitations of traditional antibiograms, clinicians have used more advanced antibiograms, including combination, syndromic, and weighted incidence syndromic antibiograms (WISCA). Combination antibiograms are useful in determining combined empiric antibacterial regimens for multidrug-resistant pathogens and are relatively easy to complete. An example of a combination antibiogram output would be the likelihood that *P. aeruginosa* will test susceptible to ≥1 agent in the FEP and tobramycin combination. The role of combination antibiograms in empiric antibacterial therapy selection has been evaluated in several published studies [19,20]. Puzniak et al. evaluated single-agent susceptibility rates for 11,701 *P. aeruginosa* isolates [20]. The susceptibility ranged from 72.7% for fluoroquinolones to 85.0% for TZP. Adding an aminoglycoside to the fluoroquinolone or TZP regimen resulted in a higher susceptibility. Syndromic antibiograms increase the likelihood of providing an effective empiric therapy for a specific infectious syndrome and may be further stratified based on the hospital location [11]. An example of a syndromic antibiogram is one that reports the susceptibility of *P. aeruginosa* to FEP among respiratory specimens from patients in the ICU at the time of culture collection. Klinker and colleagues compared antibacterial susceptibilities using a traditional versus syndromic antibiogram for common gram-negative pathogens associated with pneumonia stratified by patient location [11]. The traditional antibiogram included the susceptibility of *Escherichia coli, Klebsiella* spp., and *P*. *aeruginosa* from all sources. The syndromic antibiogram included the susceptibility for the same three gram-negative pathogens isolated from a respiratory source. The traditional antibiogram demonstrated that the susceptibilities of *E. coli* and *Klebsiella* spp. were near or greater than 90% for FEP, TZP, and MEM. Comparable antibacterial susceptibilities were not achieved for *P. aeruginosa*. When antibacterial susceptibilities were stratified by location, a 5 to 8% reduction in aggregate susceptibility for the tested antibacterial therapy was observed for isolates obtained from patients in the emergency department versus the ICU. The percent of empiric susceptibility decreased further when only *P. aeruginosa* isolates from patients in the ICU were evaluated. In contrast, a ≥90% susceptibility to ceftolozane/tazobactam (C/T) and imipenem/relebactam was maintained regardless of the isolated pathogen and/or location. Finally, WISCAs integrate patient variables, provide empiric antibacterial therapy recommendations for a specific infectious syndrome, and can be incorporated into the electronic healthcare record. For example, a WISCA antibiogram reports the susceptibility of *P. aeruginosa* to FEP among respiratory specimens from male patients ≥65 years of age with heart failure in the ICU. Ridgway and colleagues evaluated the impact of WISCA use for empiric antibacterial therapy on the hospital length of stay at four hospitals [21]. Antimicrobial stewardship physicians used WISCA in combination with clinical guidelines to provide empiric antibacterial therapy recommendations. The authors concluded that there were no overall differences in outcomes, including the length of stay, 30-day mortality, and 30-day readmission, among the intervention and control groups. Although the study failed to demonstrate a significant difference in outcomes, there were notable limitations. There was a high frequency of agreement between antibacterial stewardship physicians and primary prescribers within the intervention group. Secondly, the recommendation acceptance was low, potentially mitigating any potential benefit. Finally, approximately 90% of patients were admitted to general hospital wards and may have been less susceptible to suboptimal outcomes associated with effective antibacterial therapy delays. There are disadvantages associated with advanced antibiograms, including the fact that they are less easily understood by prescribers, often require manual completion, and lack correlation with clinical and microbiologic outcomes [11].

## 4. Novel Antibiograms to Determine Earlier Susceptibility Testing for New β-Lactam/β-Lactamase Inhibitors

As previously described, DTR pathogens may negatively impact clinical outcomes, especially amongst critically ill patients. Outcomes are driven in part by the timeliness of an appropriate empiric antibacterial therapy, which is often based on a traditional antibiogram. Novel approaches to traditional antibiograms are strategies that utilize syndromic antibiograms and include the resistance frequency amongst commonly encountered resistant pathogens and MIC data. These novel approaches may assist clinicians with antimicrobial stewardship initiatives that inform protocol or pathway development in order to better define a patient population more likely to be infected with resistant pathogens. These patients may benefit from a broader empiric coverage and/or trigger a more rapid modification of therapy. Further, an improvement in patient stratification by the frequency of resistant pathogens may highlight a cohort of patients who would benefit from an early susceptibility testing of newer agents.

Klinker and colleagues used data from a large surveillance program involving 20 institutions over a four-year period (2016–2019) to evaluate the impact of a “15% resistance frequency rule” applied to carbapenem-resistant *P. aeruginosa* and ESBL-producing *E*. *coli* and *Klebsiella pneumoniae* on achieving an empiric antibacterial susceptibility threshold ≥90% for FEP, TZP, MEM, C/T, and imipenem/relebactam in isolates from critically ill patients with pneumonia [22]. Resistance phenotypes were categorized based on the resistance to MEM (MIC ≥4 mg/L) for *P. aeruginosa* or to ceftriaxone (MIC ≥2 mg/L) for *E. coli* and *K. pneumoniae*.

After applying the “15% resistance frequency rule” to each institution that contributed isolates to the Study for Monitoring Antimicrobial Resistance Trends (SMART) surveillance program, four scenarios were identified (Table 1). In the best-case scenario (Group 1), the aggregate susceptibility was ≥90% for all antibacterial agents when the resistance frequency was ≤15% for both phenotypes. These data support the empiric use of first-line β-lactams targeting *P. aeruginosa* and ESBL *E. coli* and *Klebsiella* spp. In contrast, when the 15% resistance frequency for either phenotype was exceeded (Groups 2 or 3), the FEP and TZP susceptibility was reduced by 6 to 11% and 4 to 7%, respectively, resulting in an inability to achieve the empiric susceptibility threshold. Finally, when the resistance frequency exceeded 15% for both phenotypes (Group 4), the aggregate susceptibility declined to 77.3%, 79.3%, and 86.2% for FEP, TZP, and MEM, respectively. In this scenario, an earlier susceptibility testing for new β-lactam/β-lactamase inhibitor combinations may be warranted in order to achieve adequate empiric susceptibility rates. In support of this approach, regardless of the frequency of resistant phenotypes, C/T and imipenem/relebactam maintained an aggregate susceptibility above the ≥90% empiric susceptibility recommendation. The authors concluded that the stratification of patients by the “15% resistance frequency rule” could serve as an important decision point in supporting earlier susceptibility testing or modifying empiric therapy for respiratory tract infections to include newer therapies while awaiting final microbiology results.

**Figure 1:**
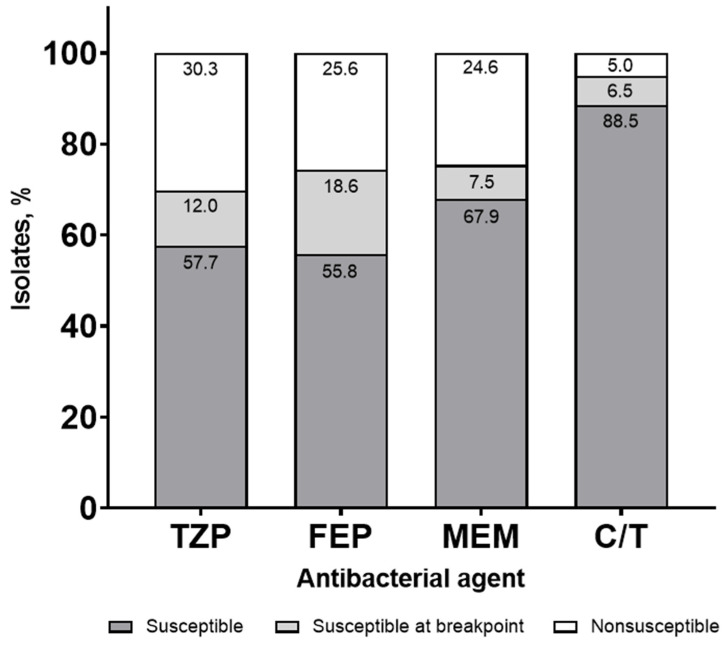
Syndromic antibiogram evaluating susceptibility for *Pseudomonas aeruginosa*, including isolates susceptible at the minimum inhibitory concentration (MIC) breakpoint collected from a blood or respiratory source. TZP, piperacillin/tazobactam; FEP, cefepime; MEM, meropenem; C/T, ceftolozane/tazobactam.

Bauer et al. evaluated the antibacterial susceptibilities for commonly used β-lactams against *P. aeruginosa* in a syndromic antibiogram, incorporating MIC distributions [23]. Due to the frequency of the baseline resistance and the challenges in achieving adequate pharmacokinetics/pharmacodynamics in critically ill patients, clinicians may be concerned with relying on certain antibacterial agents when the MIC is at the susceptible breakpoint. Similar to the study by Klinker and colleagues, an empiric antibacterial susceptibility threshold of ≥90% was targeted. A total of 3648 *P. aeruginosa* isolates, including 2500 from a blood or respiratory source, were evaluated. The traditional antibiogram demonstrated that susceptibilities for FEP, TZP, and MEM were all below the 90% threshold for *P. aeruginosa*; C/T maintained the empiric susceptibility target. Compared with the traditional antibiogram, FEP, TZP, and MEM susceptibilities were 2 to 4% lower for the syndromic antibiogram; C/T maintained a ≥90% susceptibility. Further stratification of the syndromic antibiogram by ICU admission resulted in a 6 to 8% susceptibility reduction for FEP, TZP, and MEM compared with the traditional antibiogram. In contrast, C/T maintained a ≥90% susceptibility. Upon further refinement, the syndromic antibiogram was evaluated with the incorporation of isolates categorized as susceptible, susceptible at the MIC breakpoint, and nonsusceptible. Susceptible isolates with MICs at the breakpoint were observed at 18.6%, 12.0%, 7.5%, and 6.5% for FEP, TZP, MEM, and C/T, respectively (Figure 1).

**Figure 2:**
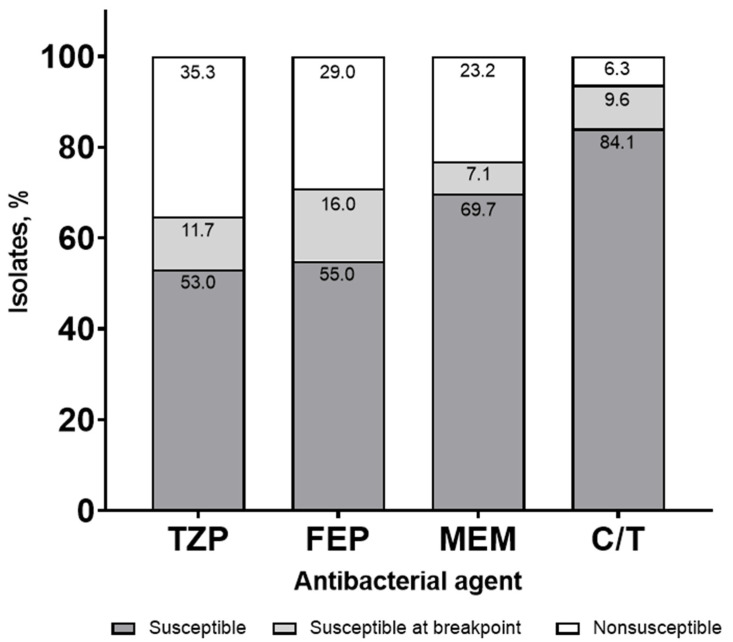
Syndromic antibiogram evaluating susceptibility for *Pseudomonas aeruginosa*, including isolates susceptible at the minimum inhibitory concentration (MIC) breakpoint collected from a blood or respiratory source stratified by ICU. TZP, piperacillin/tazobactam; FEP, cefepime; MEM, meropenem; C/T, ceftolozane/tazobactam.

Susceptibilities were lower when stratified by ICU status (64.7%, 71.0%, 76.8%, and 93.7% for TZP, FEP, MEM, and C/T, respectively), with a similar frequency of susceptibility for the breakpoint isolates (Figure 2).

The authors concluded that first-line antipseudomonal β-lactams had susceptibility rates that were lower than the recommended target when evaluating a syndromic antibiogram and incorporating MIC distributions. This type of antibiogram also supports the approach of considering susceptibility testing earlier for new β-lactam/β-lactamase inhibitor combinations.

## 5. Conclusions

The time to effective antibacterial therapy is critical, as delays in TTET have been associated with increases in mortality, especially among critically ill patients. With the traditional timeline, patients prescribed first-line β-lactams for gram-negative pathogens that were subsequently found to be DTR may not receive appropriate therapy for several days, potentially leading to increased morbidity and mortality. There is a need to identify earlier in a patient’s treatment course when the susceptibility of a new β-lactam/β-lactamase inhibitor should be assessed and reported to clinicians to determine if a therapy switch is appropriate. Commonly used antibiograms, including traditional and combination versions, may not adequately assist clinicians in making these decisions. Novel antibiograms such as syndromic antibiograms that incorporate resistant gram-negative phenotypes and/or MIC distributions may assist in determining the need for earlier susceptibility testing or the selective reporting of these results to help define an earlier optimal use of agents such as the new β-lactam/β-lactamase inhibitors.

## Figures and Tables

**Table 1:** Syndromic antibiogram evaluating resistant phenotypes, including CRPA and ESBL-E.

Group	FEP	TZP	MEM	C/T	IMR
1: CRPA and ESBL-E ≤ 15%	94.5	90.8	97.8	97.6	99.2
2: CRPA ≤ 15% and **ESBL-E > 15%**	83.3	86.7	95.9	94.5	99.2
3: **CRPA > 15%** and ESBL-E ≤ 15%	88.4	83.4	88.0	96.0	95.9
4: **CRPA and ESBL-E > 15%**	77.3	79.3	86.2	93.0	95.9

CRPA, carbapenem-resistant *Pseudomonas aeruginosa*; ESBL-E, extended-spectrum β-lactamase-producing *Escherichia coli* and *Klebsiella* spp.; FEP, cefepime; TZP, piperacillin/tazobactam; MEM, meropenem; C/T, ceftolozane/tazobactam; IMR, imipenem/relebactam. Shaded cells represent a susceptibility rate ≤90%. Bold text indicates differences from Group 1.

## Data Availability

Merck Sharp & Dohme LLC, a subsidiary of Merck & Co., Inc., Rahway, NJ, USA’s data sharing policy, including restrictions, is available at http://engagezone.msd.com/ds_documentation.php. Requests for access to the study data can be submitted through the EngageZone site or via email to dataaccess@merck.com.
